# Pulsed light epithelium-off accelerated corneal collagen crosslinking with 30mW/cm^2^ irradiance and 7.2 J/cm^2^ radiant exposure: 2-year results

**DOI:** 10.1007/s10792-025-03512-7

**Published:** 2025-04-16

**Authors:** Max Luckmann, Myriam Böhm, Eva Hemkeppler, Thomas Kohnen

**Affiliations:** Department of Ophthalmology, Goethe University, Theodor-Stern-Kai 7, 60590 Frankfurt, Germany

**Keywords:** Keratoconus, Accelerated crosslinking, Pulsed light ultraviolet-A, High-fluence

## Abstract

**Purpose:**

To evaluate the 2-year outcomes of pulsed light epithelium-off accelerated corneal collagen crosslinking (aCXL) using 30 mW/cm^2^ for 8 min (7.2 J/cm^2^) for the treatment of progressive keratoconus.

**Methods:**

A total of 23 eyes of 18 patients with progressive keratoconus, that were treated with epithelium-off pulsed light aCXL (30 mW/cm^2^, 8 min) were included in this retrospective study. Tomographic measurements and corrected distance visual acuity (CDVA) were analyzed at the baseline visit (before the aCXL treatment), and at 3-, 12- and 24-months visit.

**Results:**

The two-year results demonstrated a stable anterior flat keratometry (K1), steep keratometry (K2) and mean keratometry (K_mean_) of the anterior and posterior cornea with no significant changes at 3, 12 and 24 months postoperatively, compared to the baseline visit (*p* > 0.05). Maximal anterior keratometry (K_max_) stabilized from 60.18 ± 6.32 D at baseline to 60.04 ± 7.36 D at 24 months post-aCXL (*p* = 0.88). The keratoconus indices and astigmatism of the front and back surface of the cornea also showed stable results. The CDVA improved from 0.69 ± 0.29 logarithm of the minimum angle of resolution (logMAR) at baseline to 0.35 ± 0.21 logMAR at 24 months postoperatively (*p* = 0.16).

**Conclusion:**

Pulsed light epithelium-off accelerated CXL using 30 mW/cm^2^ for 8 min (7.2 J/cm^2^) appears to be an effective treatment modality halting progression of keratoconus even after two years.

## Introduction

Keratoconus is a degenerative disorder of the eye showing progressive corneal ectasia with increasing irregular astigmatism accompanied by corneal thinning and possible scarring of the apical part or hydrops, according to Rabinowitz et al. [1].

If keratoconus progresses, it may ultimately end in corneal transplantation, according to Seitz et al. [2]. The etiology of keratoconus seems to be multifactorial and is not yet fully understood. However, it seems to have genetic and environmental components.

The only relatively new treatment that aims to slow or halt the progression of keratoconus or other ectatic disease, e.g., pellucid marginal degeneration and after corneal refractive surgery, is corneal collagen crosslinking (CXL), which includes the use of Riboflavin (Vitamin B2) drops as photosensitizer onto the cornea followed by ultraviolet-A (UV-A) light exposure.

According to the standard or “Dresden” protocol (C-CXL), the treatment includes removal of the corneal epithelium, followed by instillation of Riboflavin eye drops for 30 min, then, continuous irradiation of the cornea for 30 min under continuation of the Riboflavin instillation at an irradiance of 3 mW/cm^2^ for 30 min, reaching a total fluence of 5.4 J/cm^2^. The treatment duration can vary from few minutes until one hour according to the set protocol which can include alteration of the duration of the Riboflavin instillation and irradiation, while the application and takes, depending on the protocol and the crosslinking procedure applied, between a few minutes and one hour [3].

CXL leads to an increased corneal rigidity and stiffens the anterior corneal stroma, thereby halting keratoconus progression and has been labelled safe due to many positive study results [4–8]. However, due to the long irradiation time of the C-CXL protocol, the problems of increased risk of infection and drying out of the cornea and the stress for both the patient and the surgeon may occur, thus, accelerated protocols (aCXL) arose which present with a shorter irradiance time and higher UV-A intensity [9].

The purpose of this study was to evaluate the clinical outcomes of pulse-light epithelium-off aCXL at 30 mW/cm^2^ for 8 min (7.2 J/cm^2^) at patients with progressive keratoconus with a 24-month follow-up (Tables 1, and 2).

**Table 1:** Tomographic parameters over a two-year period after pulsed light epithelium-off accelerated crosslinking of 30 mW/cm^2^ for 8 min with total fluence of 7.2 J/cm^2^ for the treatment of progressive keratoconus

Parameters		Baseline	3 months	12 months	24 months
K1 flat front (diopters)	N	23	23	19	23
	Mean ± Standard deviation	48.14 ± 5.30	48.67 ± 5.43	48.57 ± 5.91	48.20 ± 5.26
	*p*-value	–	0.21	0.88	0.86
K2 steep front (diopters)	N	23	23	19	23
	Mean ± Standard deviation	52.22 ± 6.04	53.07 ± 6.24	52.71 ± 6.5	52.32 ± 6.39
	*p*-value	–	0.061	0.85	0.83
Kmean front (diopters)	N	23	23	19	23
	Mean ± Standard deviation	50.07 ± 5.51	50.78 ± 5.66	50.52 ± 6.01	50.15 ± 5.61
	*p*-value	–	0.1	0.87	0.83
Kmax front (diopters)	N	23	23	19	23
	Mean ± Standard deviation	60.18 ± 6.32	61.31 ± 6.85	60.58 ± 6.73	60.04 ± 7.37
	p-value	–	0.07	0.87	0.88
Astigmatism front	N	23	23	19	23
	Mean ± Standard deviation	4.08 ± 2.73	4.41 ± 2.77	4.21 ± 3.04	4.12 ± 3.24
	*p*-value	–	0.01	0.70	0.81
Axis front	N	23	23	19	23
	Mean ± Standard deviation	104.29 ± 40.2	91.03 ± 43.97	97.62 ± 42.58	97.08 ± 34.12
	*p*-value	–	0.16	0.83	0.18
K1 flat back	N	23	23	19	23
	Mean ± Standard deviation	− 7.08 ± 1.13	− 7.17 ± 1.12	− 7.29 ± 1.15	− 7.12 ± 1.09
	*p*-value	–	0.21	0.5	0.54
K2 steep back	N	23	23	19	23
	Mean ± Standard deviation	− 7.99 ± 1.9	− 8.03 ± 1.12	− 8.04 ± 1.22	− 8.0 ± 1.14
	*p*-value	–	0.54	0.95	0.95
Kmean back	N	23	23	19	23
	Mean ± Standard deviation	− 7.49 ± 1.12	− 7.58 ± 1.09	− 7.64 ± 1.16	− 7.51 ± 1.08
	*p*-value	–	0.11	0.67	0.7
Astigmatism back	N	23	23	19	23
	Mean ± Standard deviation	0.89 ± 0.64	0.87 ± 0.54	0.75 ± 0.56	0.88 ± 0.65
	*p*-value	–	0.81	0.33	0.95
Axis back	N	23	23	19	23
	Mean ± Standard deviation	86.32 ± 36.89	84.44 ± 37.57	85.82 ± 49.03	91.58 ± 43.03
	*p*-value	–	0.50	0.90	0.57

K1, flat keratometry; K2, steep keratometry; Kmean, mean keratometry; Kmax, maximum keratometry; Ast, topographic astigmatism; D, diopters; Pre OP, preoperatively

*p*-value: statistical significance in the analysis of variances. *P*-value level set at 0.05

**Table 2:** Tomography-derived keratoconus indices over a two-year period after pulsed light epithelium-off accelerated crosslinking of 30 mW/cm^2^ for 8 min with total fluence of 7.2 J/cm^2^ for the treatment of progressive keratoconus

Parameters		Baseline	3 months	12 months	24 months
ISV	N	23	23	19	23
	Mean ± Standard deviation	113 ± 36.64	121.48 ± 35.77	110.63 ± 29.34	111.04 ± 35.64
	*p*-value	–	0.004	0.47	0.15
IVA	N	23	23	19	23
	Mean ± Standard deviation	1.18 ± 0.44	1.29 ± 0.43	1.14 ± 0.31	1.17 ± 0.44
	*p*-value	–	0.001	0.83	0.79
KI	N	23	23	19	23
	Mean ± Standard deviation	1.32 ± 0.17	1.35 ± 0.15	1.31 ± 0.14	1.30 ± 0.14
	*p*-value	–	0.01	0.34	0.05
CKI	N	23	23	19	23
	Mean ± Standard deviation	1.08 ± 0.07	1.09 ± 0.07	1.08 ± 0.07	1.08 ± 0.07
	*p*-value	–	0.11	0.79	0.93
IHA	N	23	23	19	23
	Mean ± Standard deviation	40.58 ± 29.68	35.63 ± 21.48	35.71 ± 20.21	35.51 ± 24.42
	*p*-value	–	0.49	0.25	0.48
IHD	N	23	23	19	23
	Mean ± Standard deviation	0.17 ± 0.08	0.19 ± 0.07	0.17 ± 0.07	0.17 ± 0.09
	*p*-value	–	0.02	0.48	0.43
Rmin	N	23	23	19	23
	Mean ± Standard deviation	5.66 ± 0.59	5.57 ± 0.62	5.64 ± 0.62	5.7 ± 0.67
	*p*-value	–	0.1	0.97	0.63

ISV, index of surface variance; IVA, index of vertical asymmetry; KI, keratoconus index; CKI, center keratoconus index; IHA, index of height asymmetry; IHD, index of height decentration; Rmin, minimum sagittal curvature

*p*-value, statistical significance in the analysis of variances. *P*-value level set at 0.05

## Patients and methods

### Study group

This retrospective study included 23 eyes of 18 adult patients with progressive keratoconus that received pulsed light epithelium-off aCXL of 30 mW/cm^2^ for 8 min (7.2 J/cm^2^) using KXL System (Avedro, Inc., 230 Third Avenue, Waltham, MA 02451) at the Department of Ophthalmology of Goethe-University, Frankfurt am Main. Progression of keratoconus was detected using Scheimpflug imaging (Pentacam® HR, Oculus, Wetzlar, Germany) and was defined as an increase of the maximum anterior corneal keratometry (K_max_) of 1 diopter (D) or more and/or an increase of astigmatism as determined by subjective refraction of the cornea of 1 D or more within 12 months [2, 10, 11]. Moreover, only corneas with a minimum corneal thickness of 400 μm at the thinnest point underwent the treatment [2], and only patients > 18 years of age were included in the study. Data was collected preoperatively, as well as 3, 12 and 24 months postoperatively. At 12 months 4 patients (n = 4) did not show up for their follow-up examinations, consequently leading to a smaller sample size at this time-point with 19 eyes, respectively. All patients of our study completed the 24-month follow up.

Exclusion criteria were prior ocular surgery or another eye disorder that have an influence on corneal curvature, such as prior corneal dystrophies, corneal infections, pterygium, corneal scarring, severe atopy, and pregnancy/breastfeeding.

The study presents long-term results of 23 keratoconus eyes of 18 adult patients, and changes in visual acuity, K-values, astigmatism, densitometry of the anterior and posterior surface of the cornea, keratoconus indices and central as well as thinnest corneal thickness.

For this study the Ethics approval was given by the Ethics Committee of the Goethe University Frankfurt (Ethics approval number 375/17).

### Outcome parameters

Patients underwent evaluation of their visual acuity measured in logarithm of the minimum angle of resolution (logMAR), including uncorrected distance visual acuity (UDVA) and corrected distance visual acuity (CDVA), slit lamp inspection, tonometry measurement, fundoscopy and Scheimpflug tomography (Pentacam ® HR) at all their visits. Evaluated parameters of the Pentacam ® HR measurements were: Anterior and posterior flat (K1), steep (K2), mean (K_mean_) and maximal keratometry (K_max_) values, anterior and posterior astigmatism, central corneal thickness (CCT), CT at the thinnest point, keratoconus indices, including: Index of surface variance (ISV), Index of vertical asymmetry (IVA), keratoconus index (KI), Central keratoconus index (CKI), Index of height asymmetry (IHA), Index of height decentration (IHD) and Minimal sagittal curvature (R_min_), and corneal densitometry (0–2 mm, 2–6 mm, 6–10 mm, 10–12 mm and total). Densitometry was measured using Grayscale units (GSU) using Scheimpflug tomography (Pentacam.® HR) [11].

### Surgical technique

All procedures were performed by the same surgeon (TK) using the pulsed light epithelium-off aCXL protocol of 30 mW/cm^2^ for 8 min (7.2 J/cm^2^) was used to perform the surgery. In comparison to previously used devices for CXL, which work with Gaussian beam profiles, Avedro’s KXL system uses a 9 mm beam diameter with a homogenous beam intensity profile [10], resulting in a more even distribution of the UV-A light energy across the cornea.

After applying topical anaesthetic eye drops (Proparacaine-POS® 0.5% eye drops, active substance: proxymetacainhydrochlorid, URSAPHARM Arzneimittel GmbH, Saarbrücken, Germany) a speculum was inserted. Standard hygienic protocols were followed to perform the debridement of the epithelium and Riboflavin (VibeX Rapid™: 0.1% Riboflavin, hydroxypropyl methylcellulose, Avedro Inc., 230 Third Avenue, Waltham, MA 02451) was instilled on the exposed corneal stoma. Riboflavin was reapplied every 2 min to completely cover and saturate the stoma for a total of 10 min, followed by rinsing with a balanced salt solution (BSS®, balanced salt solution, Alcon AG, Vernier-Geneva, Switzerland) before starting irradiation. Then, the pulsed (1 s (s) “on”, 1 s “off”) UV-A treatment at 30 mW/cm^2^ for a total of 8 min UV-A time was initiated, at a total fluence of 7.2 J/cm^2^ was performed. During the entire UV-A treatment, the cornea was saturated with Riboflavin as needed. After completion of treatment, topical corticosteroid (Dexa EDO® eye drops, active substance: dexamethasone, Bausch&Lomb, Vaughan, Ontario, Canada), and antibiotic eye drops without preservatives (Floxal EDO® eye drops, active substance: ofloxacin, Bausch&Lomb, Vaughan, Ontario, Canada) were administered and a bandage soft contact lens was placed on the cornea and removed a few days later, when reepithelialization was completed.

Postoperatively, the patients received standard care with the previously mentioned topical antibiotic and corticosteroid eye drops without preservatives, each 3 times daily for 4 days, followed by a topical corticosteroid treatment including Efflumidex® eye drops, (active substance: fluorometholone, Abbvie, North Chicago, Illinois, USA), 4 times daily for 1 week, followed by weekly reduction of 1 drop per week thereafter. Additionally, patients received a lubricant eye medication (Cellufresh® eye drops, active substance: carboxy-methylcellulose sodium, Allergan Inc., Irvine, California, USA), 4 times daily for 4 weeks. Postoperative follow-up examinations were performed daily from day 1 to day 4 until re-epithelialization was complete. The course of re-epithelialization proceeded without any complications in all treated eyes.

Early postoperative follow-up examination within the first four days were performed for inspection of possible haze formation, sterile infiltrates, signs of infection and ocular hypertension. Haze evaluation was based on slit lamp biomicroscopy findings and the Fantes anterior stromal haze scale [12].

### Statistical analysis

Data was collected using Microsoft Excel (Version 14.2.0, Microsoft, Redmond, Washington, USA) and statistical analysis was performed using IBM SPSS Statistics (Version 26, IBM, Armonk, New York, USA). All descriptive data are presented as mean values and standard deviations (± SD) and tested for normal distribution. A paired samples t-test was performed if the data was normally distributed, and a Wilcoxon test was performed if not normally distributed. Significance level was set at *p* < 0.05. A power calculation was conducted using Power Analysis and Sample Size Software (PASS, NCSS LLC, Kaysville, Utah, USA) using the actual results of the standard deviation of about 4.5 D of the difference in the K_max_ value from preoperatively to 2 years postoperatively and a determined power of at least 70%, 80%, and 90% for the sum of n = 23 patients’ eyes occurred for a mean difference of at least 2.4, 2.8, and 3.1 D, respectively.

## Results

The study included 23 eyes of 18 adult patients with mean age of 30.13 ± 8.75 (21–55) years. Of the 23 eyes, 19 completed the 12 months follow-up and all completed the two-year follow-up.

The UDVA showed no statistically significant change from 0.73 ± 0.34 logMAR preoperatively to 0.95 ± 0.1 logMAR (*p* = 0.1) and 0.80 ± 0.28 logMAR (*p* = 0.18) at 12 and 24 months respectively. The CDVA improved gradually without statistical significance from 0.68 ± 0.29 logMAR preoperatively to 0.55 ± 0.31 logMAR 12 months (*p* = 0.4) and 0.35 ± 0.21 logMAR 24 months (*p* = 0.16) postoperatively. The changes in visual acuity measurements over the two-year period (UDVA, CDVA) are shown in Table 3.

**Table 3:** Visual acuity over a two-year period after pulsed light epithelium-off accelerated crosslinking of 30 mW/cm^2^ for 8 min with total fluence of 7.2 J/cm^2^ for the treatment of progressive keratoconus

Parameters		Baseline	3 months	12 months	24 months
UDVA (logMAR)	N	17	20	14	21
	Mean ± Standard deviation	0.73 ± 0.34	0.83 ± 0.24	0.95 ± 0.1	0.8 ± 0.28
	*p*-value	–	0.76	0.62	0.18
CDVA (logMAR)	N	20	23	19	22
	Mean ± Standard deviation	0.68 ± 0.29	0.46 ± 0.24	0.55 ± 0.31	0.18 ± 0.21
	*p*-value	–	1	0.4	0.16

UDVA, uncorrected distance visual acuity; CDVA, corrected distance visual acuity; logMAR, logarithm of the minimum angle of resolution

*p*-value: statistical significance in the analysis of variances. *P*-value level set at 0.05

Keratometry as well as astigmatism and axis for both the anterior corneal curvature (ACC) and the posterior cornea curvature (PCC) showed no progression after 2 years. Anterior K_mean_ remained relatively stable, changed from 50.07 ± 5.51 to 50.52 ± 6.01 12 months postoperatively (*p* = 0.88) to 50.15 ± 6.61 D 24 months postoperatively (*p* = 0.833). Posterior K_mean_ changed from − 7.49 ± 1.12 to − 7.64 ± 1.16 12 months after surgery (*p* = 0.67) to − 7.51 ± 1.08 D (*p* = 0.70). K_max_ showed no statistically significant change from 60.18 ± 6.32 D preoperatively to 60.04 ± 7.37 D at 24 months postoperatively (*p* = 0.88). No statistically significant changes were observed 24 months postoperatively. K-values, astigmatism and axis changes of the ACC and PCC of the cornea overtime are shown in Table 1.

**Fig. 1:**
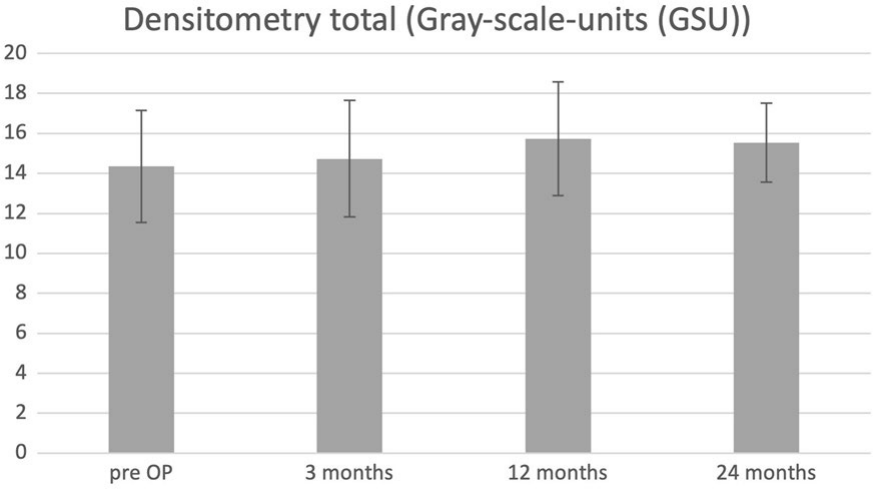
Total densitometry in Gray-scale-units (GSU) development changes over two years after pulsed light epithelium-off accelerated crosslinking of 30 mW/cm^2^ for 8 min with total fluence of 7.2 J/cm^2^ for the treatment of progressive keratoconus

**Fig. 2:**
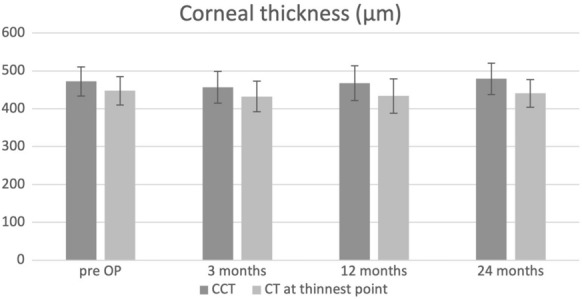
Central corneal thickness and corneal thickness at thinnest point (µm) development changes over two years after pulsed light epithelium-off accelerated crosslinking of 30 mW/cm^2^ for 8 min with total fluence of 7.2 J/cm^2^ for the treatment of progressive keratoconus

Corneal thickness at the center of the cornea and the thinnest point of the cornea remained stable 24 months after surgery. CCT changed from 472.26 ± 38.33 to 478.85 ± 41.2 µm 24 months postoperatively (*p* = 0.90). CT at the thinnest point changed from 447.83 ± 37.51 to 440.57 ± 36.58 µm 24 months after surgery (*p* = 0.16). All densitometry values slightly increased without statistical significance, except densitometry 6–10 mm (*p* = 0.03). Total densitometry increased from 14.35 ± 2.81 to 15.54 ± 1.97 Grayscale units (GSU) 24 months postoperatively (*p* = 0.08). Densitometry and corneal thickness values (Densitometry (0–2 mm, 2–6 mm, 6–10 mm, 10–12 mm and total as well as CCT, CT at thinnest point,) are shown in Figs. 1, 2 and Table 4.

**Table 4:** Densitometry values of the cornea measured in Gray scale units (GSU) and corneal thickness in µm over a two-year period after pulsed light epithelium-off accelerated crosslinking of 30 mW/cm^2^ for 8 min with total fluence of 7.2 J/cm^2^ for the treatment of progressive keratoconus

Parameters		Baseline	3 months	12 months	24 months
CCT	N	23	23	19	22
	Mean ± Standard deviation	472.26 ± 38.33	456.61 ± 42.09	467.37 ± 45.83	473.86 ± 41.17
	*p*-value	–	0.01	0.76	0.9
TCT	N	23	23	19	23
	Mean ± Standard deviation	447.83 ± 37.51	432.22 ± 40.39	433.53 ± 45.24	440.57 ± 36.58
	*p*-value	–	0.001	0.53	0.16
CD 0–2 mm	N	23	23	18	23
	Mean ± Standard deviation	15.1 ± 3.69	16.81 ± 3.32	17.31 ± 3.01	16.55 ± 2.45
	*p*-value	–	0.06	0.001	0.09
CD 2–6 mm	N	23	23	18	23
	Mean ± Standard deviation	13.58 ± 2.87	14.72 ± 2.76	15.71 ± 3.86	14.59 ± 1.72
	*p*-value	–	0.08	0.002	0.17
CD 6–10 mm	N	23	23	18	23
	Mean ± Standard deviation	12.91 ± 3.17	12.82 ± 3.04	14.33 ± 4.11	14.09 ± 3.13
	*p*-value	–	0.17	0.02	0.03
CD 10–12 mm	N	23	23	18	23
	Mean ± Standard deviation	19.44 ± 7.7	17.51 ± 5.94	18.51 ± 5.05	19.77 ± 4.22
	*p*-value	–	0.24	0.55	0.48
CD Total	N	23	23	18	23
	Mean ± Standard deviation	14.35 ± 2.81	14.73 ± 2.92	15.73 ± 2.84	15.54 ± 1.97
	*p*-value	–	0.48	0.01	0.08

CCT, central corneal thickness; TCC, thinnest corneal thickness; CD, Corneal densitometry; CT, total densitometry

*p*-value: statistical significance in the analysis of variances. *P*-value level set at 0.05

Changes in keratoconus indices (ISV, IVA, KI, CKI, IHA, IHD, R_min_) are shown in Table 2.

The most interesting change was notable for IHA, which decreased from 40.58 ± 29.68 to 35.51 ± 24.42 24 months postoperatively, though without statistical significance (*p* = 0.48). Additionally, our data showed a statistically significant change in ISV 113 ± 26.64 to 121.48 ± 35.77 (*p* = 0.004), IVA 1.18 ± 0.44 to 1.29 ± 0.43 (*p* = 0.001) and KI 1.32 ± 0.17 to 1.35 ± 0.15 (*p* = 0.011) at 3 months postoperatively but no statistically significant change at 24 months postoperatively, except for KI, which decreased to 1.30 ± 0.14 (*p* = 0.045) 2 years after surgery.

Slit lamp inspections in the first four days postoperatively showed no infections, no infiltrates, and no intraocular pressure elevation (> 24 mmHg or > 10 mmHg from preoperatively). Five cases of corneal haze were reported in the first four postoperative days and eight cases were reported after more than 4 days postoperatively. All cases of corneal haze resolved over time and no haze was detected 24 months after treatment. Postoperative complications are shown in Table 4 of the supplementary material.

## Discussion

Bak-Nielsen et al. found that, in the last two decades, the incidence of keratoconus in Denmark increased twofold–threefold. It was suggested that the increase in incidence is possibly largely a consequence of more complete data due to the availability of corneal crosslinking. Moreover, other factors described include a true increased incidence rate, better diagnostic equipment resulting in diagnosis of milder non progressive cases as well as immigration from areas of higher prevalence, as observed in the Middle Eastern and Asia populations [13]. In case of a true increased incidence rate, more patients might need CXL in the future.

Keratoconus can manifest between childhood and young adult age and progression is mainly observed in adolescence and early adulthood, whereas the disease shows a more aggressive pattern in pediatric patients and adolescents, as described by Galvis et al. [14]. Collagen fibrils of the cornea become thicker, and the tissue stiffens due to natural occurrence of crosslinking in older patients. Interestingly, our study included middle aged patients with a progression of keratoconus, and although this is not frequent, keratoconus progression according to the defined progression criteria of our study was observed in two patients that were older than 40 years [2, 10].

Few studies with a long-term follow-up of pulsed light aCXL exist, particularly regarding the anterior and posterior surface of the cornea. Mazzotta et al. [15] evaluated the treatment with pulsed light aCXL at 15 mW/cm^2^ with a fluence of 5.4 J/cm^2^ at a large cohort of 132 eyes until two-years postoperatively. Their data suggests that this protocol is a safe procedure, can stabilize keratoconus progression even after two years, and improve CDVA significantly (from 0.27 ± 0.14 to 0.14 ± 0.14 logMAR, *p* = 0.0023). We need to note that the protocol used included pulsed light UV-A light application which did not exceed 5.4 J/cm^2^, and the sample size was bigger compared to the present study. It was indicated that pulsed light aCXL can halt the progression of keratoconus.

Our study found no significant change in CDVA after 12 or 24 months postoperatively. The peak in CDVA at 12 months postoperatively could be attributed to the small sample size at 12 months postoperatively of only 19 eyes compared to 23 eyes at 3 months returning for examinations. Additionally, high K_max_ values preoperatively might be responsible for the lack of a significant improvement in visual acuity after 12 months postoperatively.

Ziaei et al. compared pulsed light (8 min, 1 s on, 1 s off) and continuous light (4 min) protocols with 30 mW/cm^2^ and a total energy dose of 7.2 J/cm^2^.[16] No statistically significant change was found in CDVA 1 year postoperatively, but a significant improvement was found after 2 years in both study groups. CDVA improved from 0.3 ± 0.16 to 0.23 ± 0.17 logMAR (*p* = 0.04) after pulsed CXL in pulsed light exposure and from 0.36 ± 0.22 to 0.26 ± 0.27 logMAR (*p* = 0.02) after continuous CXL in continuous light exposure. Furthermore, no statistically significant change was seen in both groups regarding K_mean_. K_max_ decreased significantly in the continuous light from 57.48 ± 5.84 to 55.73 ± 6.04 D (*p* = 0.01) two years postoperatively.

Our study showed stable keratometry values for the anterior and posterior surface of the cornea 24 months after surgery. K_mean_ front remained stable from 50.07 ± 5.51 to 50.15 ± 6.61 D (*p* = 0.83), as did K_mean_ back with − 7.49 ± 1.12 at baseline compared to − 7.51 ± 1.08 D postoperatively (*p* = 0.7). These results suggest that the aCXL protocol used in this study stabilized the cornea effectively and could halt the progression of keratoconus. In our protocol K_max_ changed from 60.18 ± 6.32 D to 60.58 ± 6.73 D 1 year after surgery and 60.04 ± 7.37 D after 24 months (*p* = 0.88). According to the Cochrane Eyes and Vision Committee a change of K_max_ 12 months after CXL ranged from − 0.5 D to + 1.99 D, with a mean difference of 0.99 D [17]. This is consistent with our results, where a mean difference of + 0.4 D 12 months post-CXL was observed.

Very limited data from other studies on the keratometry values for the posterior of the cornea exists. Although Xanthopoulou et al. used a continuous light aCXL protocol of 9 mW/cm^2^, a significant decrease in anterior flat keratometry and K_max_ 24 months postoperatively was found [18]. On the other hand, the posterior keratometry values after CXL showed a statistically significant increase > 24 months postoperatively.

This is in line with another study by Sedaghat et al. (2015) finding Ksteep, Kflat, and Kmax of the back surface significantly higher after 12 months compared with preoperative data [19].

Although not reaching statistical significance, Steinberg et al. (2014) find increasing posterior elevation values after CXL (Dresden protocol), despite a stabilized anterior corneal surface [20]. This is in line with our results showing slightly increasing K-values, however not reaching statistical significance. This might be a sign of ongoing ectatic changes in the posterior corneal surface with the posterior surface not being affected by CXL. Further studies regarding the changes of the posterior surface of the cornea after CXL are needed.

Ziaei et al. [21] used a pulsed light (1 s on, 1 s off), 45 mW/cm^2^ for the same surface dose of 7.2 J/cm^2^ in a prospective 24-month follow-up study. Their findings, like ours, support that aCXL at high irradiances is an effective procedure to treat progressive keratoconus. Keratometry, astigmatism, corneal thickness and densitometry remained stable two years after treatment in the 40 eyes enrolled in their study.

In comparison to Sherif et al. [22], where 71.4% (n = 10) of the eyes who received aCXL with a continuous light irradiance of 30 mW/cm^2^ for a total dose of 5.4 J/cm^2^ showed postoperative haze, we encountered haze in only 34.8% (n = 8) of the patients treated. All the cases of stromal haze resolved within the first year of their study whilst our cases of haze resolved within the first month postoperatively. We reported no cases of haze at the 3-month follow-up. This might suggest that the high fluence, pulsed light, epithelium-off aCXL protocol leads to an early peak in corneal haze but also to an earlier regression. These findings could also be explained by the retrospective nature of our study. Since haze occurred within the first four days after the treatment it might be argued that epithelial edema could have been mistaken for haze.

Sherif et al. [22] showed a statistically significant reduction in CCT from baseline at 6- and 12-months post operation. CCT decreased from 484.57 ± 19.45 to 469.64 ± 20 μm at 12 months postoperatively (*p* = 0.028). Sherif et al. [22] used a continuous light, 30 mW/cm^2^ (5.4 J/cm^2^) aCXL surgical technique. In comparison to their results, a significant decrease in CCT could not be found in our study. Greenstein et al. [23] suggested that the thinning in early follow-up inspections might be due to the collagen fibrils compressing or the apoptosis of keratocytes.

No significant changes were found for the keratoconus indices IVA, ISV, CKI, IHA, IHD and R_min_. In our study, KI showed a statistically significant decrease from 1.32 ± 0.17 to 1.30 ± 0.14 (*p* = 0.05). KI is the ratio between the cornea’s upper and lower half mean radius values and hence an efficient index to differentiate a healthy eye from an eye with keratoconus. The reduction we experienced in KI showed that the upper and lower half of the cornea assimilated and thus the progression of the illness seemed to be halted. The analyzed keratoconus indices showed stable values in this study and seemed to support our thesis of an effective halt of keratoconus progression two years after aCXL. Omar et al. found a significant change in ISV, IVA, and KI 12 months after epithelium-off aCXL with pulsed light (1 s on, 1 s off) and a total energy dose of 7.2 J/cm^2^ and conclude that ISV, IHA and KI can add value in follow-up examination of eyes treated with aCXL [24].

Mazzotta et al. compared one-year results of pulsed light and continuous-light aCXL and found that oxygen is essential for the collagen crosslinking reaction. They confirmed that pulsed light increases the availability of oxygen and thus generates better functional outcomes postoperatively. Therefore, pulsed light aCXL protocols seem to be superior to continuous-light aCXL protocols in terms of efficacy [25].

Wu et al. compared different CXL protocols that have evolved over time. It was found that aCXL protocols with lower UV-A illumination and longer durations provide better results, mainly due to the oxidative pathway of crosslinking [26]. In this pathway the crosslinks are induced by Riboflavin radicals or reactive oxygen species which form under UV-A light exposure. In our clinic a pulsed light aCXL protocol is used on a routine basis that may maximize oxygen diffusion and thus might provide better functional outcomes postoperatively. However, further studies are needed to prove the potential positive effect of the pulsed light protocol during our aCXL procedures. Gore et al. also used a pulsed light 1.5 s on/off aCXL protocol with 30mW/cm^2^ and a total energy of 7.2 J/cm^2^ and found it to be a safe, effective, and refractively neutral procedure [27].

The main limitation of this study is its retrospective nature. There was a dropout rate during the examination visits. At 12 months 17.4% (n = 4) of the eyes examined did not show up for their follow-up examinations. However, all 23 eyes completed the 24-month examination. Another limitation of the study is the relatively small sample size. Moreover, since the data was analyzed retrospectively the demarcation line cannot be analyzed since it was not measured during clinical routine. Lastly, the relatively high patient age, which is higher than the usual age of participants in most publications is a limitation of this study, which makes it less comparable.

In conclusion, the two-year results suggest that pulsed light epithelium-off aCXL with 30 mW/cm^2^ for 8 min (7.2 J/cm^2^) appears to halt progression of keratoconus with regards to keratometry and astigmatism of the anterior and posterior surface of the cornea, the keratoconus indices, and visual acuity. Moreover, it showed no difference in densitometry measurement two years after surgery. The data also suggests that it is a safe procedure with some short-term complications in form of corneal haze that, however, resolved within 1 month in all eyes. None of our patients experienced any side effects such as infiltrates, serious infections, or hypertension of the bulbus, which could lead to permanent loss of vision.

## Data Availability

No datasets were generated or analysed during the current study.

## References

[1] Rabinowitz YS (1998) Keratoconus. Surv Ophthalmol 42(4):297–319. 10.1016/s0039-6257(97)00119-7 9493273 10.1016/s0039-6257(97)00119-7

[2] Seitz B, Daas L, Hamon L, Xanthopoulou K, Goebels S, Spira-Eppig C, Razafimino S, Szentmáry N, Langenbucher A, Flockerzi E (2021) Stadiengerechte Therapie des Keratokonus [Stage-appropriate treatment of keratoconus]. Der Ophthalmologe: Zeitschrift der Deutschen Ophthalmologischen Gesellschaft 118(10):1069–1088. 10.1007/s00347-021-01410-8 34181061 10.1007/s00347-021-01410-8 PMC8492599

[3] Wollensak G, Spoerl E, Seiler T (2003) Riboflavin/ultraviolet-a-induced collagen crosslinking for the treatment of keratoconus. Am J Ophthalmol 135(5):620–627. 10.1016/s0002-9394(02)02220-1 12719068 10.1016/s0002-9394(02)02220-1

[4] Wollensak G, Spoerl E, Seiler T (2003) Stress-strain measurements of human and porcine corneas after riboflavin-ultraviolet-A-induced cross-linking. J Cataract Refract Surg 29(9):1780–1785. 10.1016/s0886-3350(03)00407-3 14522301 10.1016/s0886-3350(03)00407-3

[5] Kohlhaas M, Spoerl E, Schilde T, Unger G, Wittig C, Pillunat LE (2006) Biomechanical evidence of the distribution of cross-links in corneas treated with riboflavin and ultraviolet A light. J Cataract Refract Surg 32(2):279–283. 10.1016/j.jcrs.2005.12.092 16565005 10.1016/j.jcrs.2005.12.092

[6] Spoerl E, Huhle M, Seiler T (1998) Induction of cross-links in corneal tissue. Exp Eye Res 66(1):97–103. 10.1006/exer.1997.0410 9533835 10.1006/exer.1997.0410

[7] Raiskup-Wolf F, Hoyer A, Spoerl E, Pillunat LE (2008) Collagen crosslinking with riboflavin and ultraviolet-A light in keratoconus: long-term results. J Cataract Refract Surg 34(5):796–801. 10.1016/j.jcrs.2007.12.039 18471635 10.1016/j.jcrs.2007.12.039

[8] Wittig-Silva C, Chan E, Islam FM, Wu T, Whiting M, Snibson GR (2014) A randomized, controlled trial of corneal collagen cross-linking in progressive keratoconus: three-year results. Ophthalmology 121(4):812–821. 10.1016/j.ophtha.2013.10.028 24393351 10.1016/j.ophtha.2013.10.028

[9] Waszczykowska A, Jurowski P (2015) Two-year accelerated corneal cross-linking outcome in patients with progressive keratoconus. Biomed Res Int 2015:325157. 10.1155/2015/325157 25629044 10.1155/2015/325157 PMC4300022

[10] Koller T, Iseli HP, Hafezi F, Vinciguerra P, Seiler T (2009) Scheimpflug imaging of corneas after collagen cross-linking. Cornea 28(5):510–515. 10.1097/ICO.0b013e3181915943 19421048 10.1097/ICO.0b013e3181915943

[11] Asrar A, Ikram B, Khan H, Asrar M (2016) Normal values of corneal optical densitometry using pentacam scheimpflug camera. Adv Ophthalmol Vis Syst. 5(1):202–209. 10.15406/aovs.2016.05.00142

[12] Fantes FE, Hanna KD, Waring GO 3rd, Pouliquen Y, Thompson KP, Savoldelli M (1990) Wound healing after excimer laser keratomileusis (photorefractive keratectomy) in monkeys. Arch Ophthalmol 108(5):665–675. 10.1001/archopht.1990.01070070051034 2334323 10.1001/archopht.1990.01070070051034

[13] Bak-Nielsen S, Ramlau-Hansen CH, Ivarsen A, Plana-Ripoll O, Hjortdal J (2019) Incidence and prevalence of keratoconus in Denmark - an update. Acta Ophthalmol 97(8):752–755. 10.1111/aos.14082 30964230 10.1111/aos.14082

[14] Galvis V, Tello A, Ortiz AI, Escaf LC (2017) Patient selection for corneal collagen cross-linking: an updated review. Clin Ophthalmol 11:657–668. 10.2147/OPTH.S101386 28435217 10.2147/OPTH.S101386 PMC5391157

[15] Mazzotta C, Baiocchi S, Bagaglia SA, Fruschelli M, Meduri A, Rechichi M (2017) Accelerated 15 mW pulsed-light crosslinking to treat progressive keratoconus: two-year clinical results. J Cataract Refract Surg 43(8):1081–1088. 10.1016/j.jcrs.2017.05.030 28917411 10.1016/j.jcrs.2017.05.030

[16] Ziaei M, Gokul A, Vellara H, Meyer J, Patel D, McGhee CNJ (2019) Prospective two-year study of clinical outcomes following epithelium-off pulsed versus continuous accelerated corneal crosslinking for keratoconus. Clin Experiment Ophthalmol 47(8):980–986. 10.1111/ceo.13567 31170327 10.1111/ceo.13567

[17] Ng SM, Ren M, Lindsley KB, Hawkins BS, Kuo IC (2021) Transepithelial versus epithelium-off corneal crosslinking for progressive keratoconus. Cochrane Database Syst Rev 3(3):013512. 10.1002/14651858.CD013512.pub2 10.1002/14651858.CD013512.pub2 PMC8094622 33765359

[18] Xanthopoulou K, Milioti G, Daas L, Munteanu C, Seitz B, Flockerzi E (2022) Accelerated corneal crosslinking causes pseudoprogression in keratoconus within the first 6 weeks without affecting posterior corneal curvature. Eur J Ophthalmol 32(5):2565–2576. 10.1177/11206721221099257 35535408 10.1177/11206721221099257

[19] Sedaghat M, Bagheri M, Ghavami S, Bamdad S (2015) Changes in corneal topography and biomechanical properties after collagen cross linking for keratoconus: 1-year results. Middle East Afr J Ophthalmol 22(2):212–219. 10.4103/0974-9233.151877 25949080 10.4103/0974-9233.151877 PMC4411619

[20] Steinberg J, Ahmadiyar M, Rost A, Frings A, Filev F, Katz T, Linke SJ (2014) Anterior and posterior corneal changes after crosslinking for keratoconus. Optom Vis Sci: Off Publ Am Acad Optom 91(2):178–186. 10.1097/OPX.0000000000000141 10.1097/OPX.0000000000000141 24445720

[21] Ziaei M, Vellara H, Gokul A, Patel D, McGhee CNJ (2019) Prospective 2-year study of accelerated pulsed transepithelial corneal crosslinking outcomes for Keratoconus. Eye (Lond) 33(12):1897–1903. 10.1038/s41433-019-0502-3 31273313 10.1038/s41433-019-0502-3 PMC7002515

[22] Sherif AM (2014) Accelerated versus conventional corneal collagen cross-linking in the treatment of mild keratoconus: a comparative study. Clin Ophthalmol 8:1435–1440. 10.2147/OPTH.S59840 25120349 10.2147/OPTH.S59840 PMC4128847

[23] Greenstein SA, Shah VP, Fry KL, Hersh PS (2011) Corneal thickness changes after corneal collagen crosslinking for keratoconus and corneal ectasia: one-year results. J Cataract Refract Surg 37(4):691–700. 10.1016/j.jcrs.2010.10.052 21420594 10.1016/j.jcrs.2010.10.052

[24] Omar IAN, Zein HA (2019) Accelerated epithelium-off corneal collagen cross-linking for keratoconus: 12-month results. Clin Ophthalmol 13:2385–2394. 10.2147/OPTH.S232118 31824132 10.2147/OPTH.S232118 PMC6900281

[25] Mazzotta C, Traversi C, Paradiso AL, Latronico ME, Rechichi M (2014) Pulsed light accelerated crosslinking versus continuous light accelerated crosslinking: one-year results. J Ophthalmol 2014:604731. 10.1155/2014/604731 25165576 10.1155/2014/604731 PMC4137545

[26] Wu D, Lim DK, Lim BXH, Wong N, Hafezi F, Manotosh R, Lim CHL (2021) Corneal cross-linking: the evolution of treatment for corneal diseases. Front Pharmacol 12:686630. 10.3389/fphar.2021.686630 34349648 10.3389/fphar.2021.686630 PMC8326410

[27] Gore DM, Leucci MT, Koay SY, Kopsachilis N, Nicolae MN, Malandrakis MI, Anand V, Allan BD (2021) Accelerated pulsed high-fluence corneal cross-linking for progressive keratoconus. Am J Ophthalmol 221:9–16. 10.1016/j.ajo.2020.08.021 32818448 10.1016/j.ajo.2020.08.021

